# Practices and perceptions at the COVID‐19 transition in undergraduate animal science courses

**DOI:** 10.1002/nse2.20039

**Published:** 2021-02-07

**Authors:** MaryGrace Erickson, Michel A. Wattiaux

**Affiliations:** ^1^ Dep. of Animal and Dairy Sciences Univ. of Wisconsin 266 Animal Sciences Building, 1675 Observatory Dr. Madison WI 53706‐1205 USA

## Abstract

The swift transition to remote learning in response to the COVID‐19 pandemic presented substantial challenges for both students and instructors in post‐secondary natural sciences education. To examine teaching practices and student engagement during the emergency remote learning in the Spring 2020 semester, we surveyed 10 instructors and 261 students in an animal and dairy sciences department at a large midwestern university. Instructors reported using a diversity of teaching practices. On average, students perceived high teaching presence and cognitive presence and moderate social presence during emergency remote learning. Student‐reported educational experience differed substantially between courses and explained a significant amount of variance in student engagement and satisfaction outcomes (*p* < .001). Open‐ended responses revealed beliefs and attributions about remote learning that shaped students’ interpretations of educational experiences. Results support the validity of the Community of Inquiry (CoI) framework for assessing emergency remote learning and suggest future research on modulators of social presence.

**Abbreviations**
CFA: confirmatory factor analysis
CFI: comparative fit index
CI: confidence interval
CoI: community of inquiry
FIML: full‐information maximum likelihood
M: mean
RMSEA: root mean square error of approximation
SRMR: standardized root mean square residual
TA: teaching assistant
TLI: Tucker‐Lewis index
TPI: Teaching Practices Inventory.

## 1 INTRODUCTION

In the Spring 2020 semester, instructors coordinated massive efforts to adapt natural sciences courses to remote learning in response to COVID‐19 restrictions (Sutton & Jorge, 2020). Nearly 1 year later, online and remote instruction are forecasted to remain dominant undergraduate teaching modalities at many U.S. universities (The College Crisis Initiative, 2020). Although technology‐integrated pedagogies have developed substantially in natural sciences education in the past decade, the shift to remote instruction during the Spring 2020 semester occurred with unanticipated urgency and magnitude. In coping with this challenge, instructors and students formed new norms, values, and beliefs about online and remote learning that have implications for the viability of these teaching modalities in the mid‐pandemic and post‐pandemic university paradigm (Hodges, Moore, Locke, Trust, & Bond, 2020).

For many institutions, the sudden transition to remote teaching constituted a prolonged emergency. Capacity for remote teaching depends on information and communication technology infrastructure; available training, support, and funding; institutional and departmental teaching culture; student preparedness for remote learning; and faculty workload and motivation, among other factors (Knysh & Dudziak, 2020; Meyer & Xu, 2007). In past research, instructors reported that teaching online imposed a substantial workload above teaching in‐person, typically requiring weeks or months more preparation (Freeman, 2015). This suggests that during the Spring 2020 semester, instructors dedicated substantial time above their contractual obligations to adapt to emergency remote instruction. To our knowledge, research summarizing the emergency remote teaching practices used by natural sciences educators is still forthcoming.

In addition to faculty and institutional factors, student personal and social factors are critical determinants of the remote learning environment. In early reports, students described diverse personal concerns affecting their educational experience during spring 2020 emergency remote learning. Ramachandran and Rodriguez (2020) list altered living or financial conditions, difficulties focusing, technology/network issues, and mental health as common student‐reported concerns. Research showed that students from low‐income households and racial/ethnic minorities were more likely than white or high‐income students to report connectivity issues affecting their learning (Means & Neisler, 2020). Although a great deal of research suggests that online and blended learning can be as effective as in‐person instruction (Veneri & Ganotti, 2014), even for complex practical skills (McCutcheon, Alzghari, Lee, Long, & Marquez, 2017) it is unclear whether emergency remote teaching practices achieved similar positive outcomes during Spring 2020 (Jeffery & Bauer, 2020).

Natural sciences educators are in uncharted territory in the COVID‐19 world. A great deal more research is needed to understand how instructional systems responded to initial challenges, how such systems are reaching new equilibria with remote and blended learning, and how universities can continue their missions to educate, empower, and serve given the shifting educational paradigm. As a preliminary observational study, we surveyed instructors and students in an animal and dairy sciences department regarding emergency remote teaching in the Spring 2020 semester. The present study describes instructors’ emergency remote teaching practices, student perceptions of educational experience, and student outcomes related to engagement and satisfaction.

## 2 RESEARCH DESIGN AND QUESTIONS

Our research used a mixed‐method, concurrent nested design (Creswell, 2015). We surveyed 10 agriculture instructors and 261 students during the Spring 2020 semester addressing research questions in the following four categories:

1. *Perceived preparedness*. To what extent were students and instructors prepared for a sudden transition to remote learning?
2. *Remote teaching practices*. What instructor practices and priorities characterized typical remote classes?
3. *Student perceived educational experience*.
  - How did students rate their Spring 2020 experience of social presence, cognitive presence, and teaching presence in online communities of inquiry?
  - How were student perceptions of social presence, cognitive presence, and teaching presence influenced by student demographics and classroom‐level variance?
4. *Student engagement and satisfaction outcomes*. How were student outcomes of satisfaction and perceived change in engagement (relative to prior the pandemic) related to student demographics, educational experience, and classroom‐level variance?

**Core Ideas**

- Lecture‐based approaches dominated emergency remote instruction.
- Students perceived high teaching and cognitive presence and moderate social presence.
- Student‐reported educational experience varied between courses.
- Social presence predicted satisfaction and engagement outcomes.
- Student expectations, beliefs, and attributions about remote learning shaped outcomes.

**FIGURE 1:**
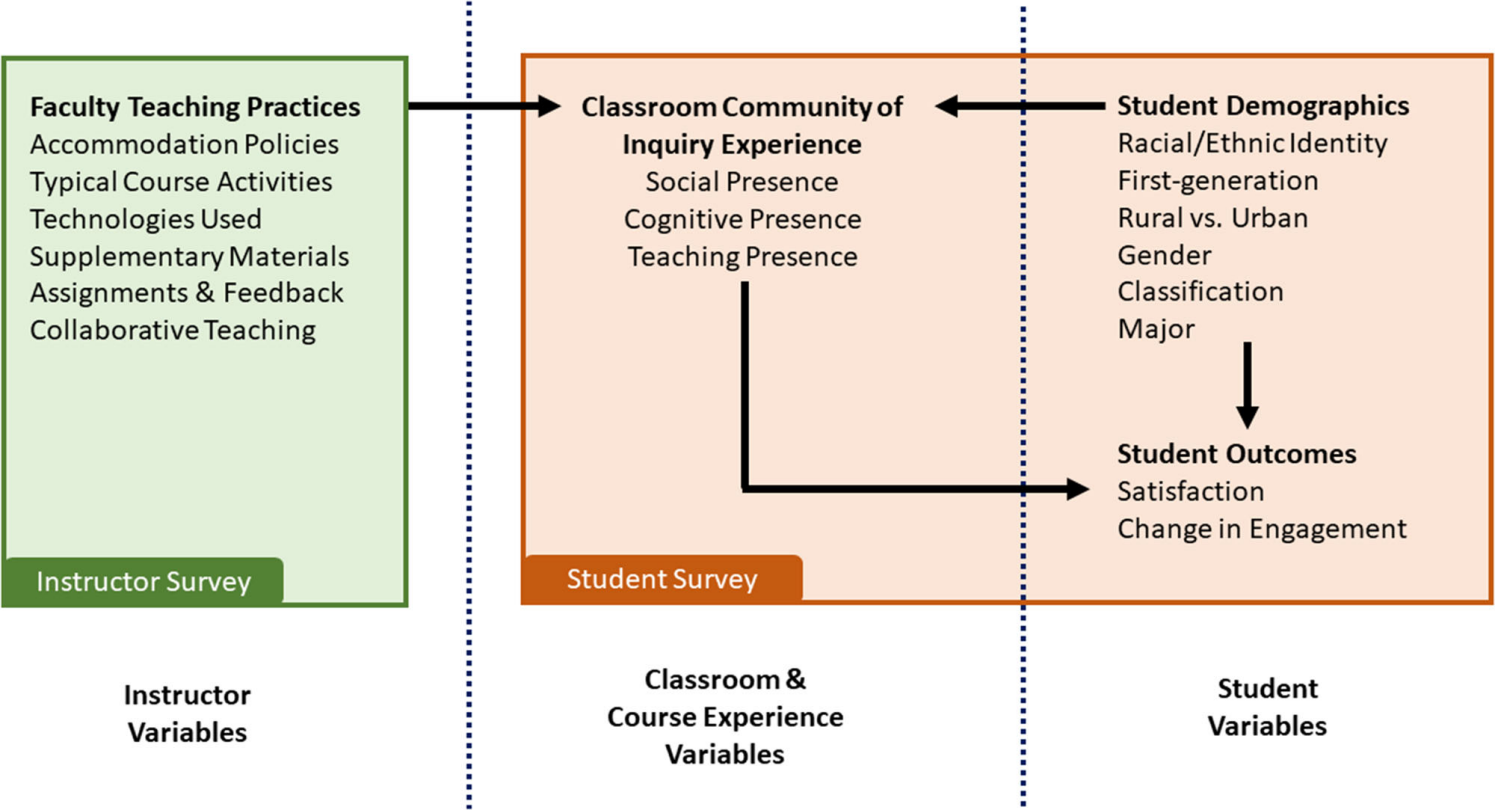
Conceptual model of instructor, course, and student variables assessed in this study

Figure 1 shows a conceptual model of proposed relationships among instructor, course, and student variables considered in the present study. Due to the small number of instructors involved in the study, our focus on instructor practices was descriptive. Our analysis focused on student‐level rather than classroom‐level variance because the low number of classes provided insufficient power to separate out classroom‐level effects. However, when considering student variables, we considered variance between courses as a proxy for differences attributable to varying learning environments and instructional practices. Additionally, we investigated relationships among student perceptions of educational experience to student personal variables including demographics and engagement and satisfaction outcomes.

## 3 METHODS

### 3.1 Context and participants

Our research took place at a large, midwestern university (Carnegie Basic classification: Doctoral Universities, Very High Research Activity). We surveyed 10 instructors of 10 mid‐ to upper‐level (150–400 level) animal science courses conducted during the spring 2020 semester, and 261 student respondents across these courses (Table 1). All instructors of all surveyed courses responded. In the single case where an instructor taught more than one course, they completed a single questionnaire applying to both courses. A course with contributions from two faculty members provided two separate instructor survey responses, which we retained. In general, student response rates were excellent. In the few cases that student response rates were poor, we retained data from instructor respondents for its value to our first two objectives.

**TABLE 1:** Distribution of student and instructor respondents across courses and course types

Course	Instructor respondents	Student respondents	Student enrollment	Student response rate
	*n*			%
A	1	114	143	79.7
B	2	46	50	92.0
C	1	42	60	70.0
D	1	26	28	92.9
E	1	24	31	77.4
F	1	23	26	88.5
G	1	18	24	75.0
H	1	4	8	50.0
I	1	2	29	6.9
J	1	1	20	5.0
Total	10	300	419	71.6

*Note*: Courses D and G were taught by one instructor who provided a single response applying to both courses. A total of 300 student responses represent 261 distinct students who took multiple surveys representing different courses in which they were enrolled.

Table 2 summarizes the demographics of student respondents. Most students identified as racial/ethnic non‐minorities and roughly a quarter reported that neither of their parents/guardians had completed a 4‐year degree. A majority identified urban communities as their familial hometowns. Females comprised nearly 75% of the sample. Students were distributed across year classifications and represented animal science, dairy science, and a variety of other majors.

**TABLE 2:** Demographics of student sample (*N* = 261)

Demographic	*N*	%
Racial/ethnic identity		
Non‐minority	201	78.2
Minority	56	21.8
Family higher education history		
Not first‐generation to obtain 4‐year degree	196	76.3
First‐generation to obtain 4‐year degree	61	23.7
Community		
Urban	159	61.9
Rural	98	38.1
Gender		
Female	182	70.8
Male	72	28.0
Non‐binary/prefer not to respond	3	1.2
Classification		
Freshman	62	24.1
Sophomore	49	19.1
Junior	70	27.2
Senior	73	28.4
Graduate/nontraditional	3	1.2
Major		
Animal science	62	24.1
Dairy science	33	12.8
Other^a^	162	63.0

^a^ Other = primarily other (non‐specified) majors in the College of Agriculture and Life Sciences based on historic course enrollment.

### 3.2 Survey procedures

The Institutional Review Board supervised all study procedures (Protocol no. 2020‐0032). All surveys were administered in the window from 23 Apr. 2020 to 10 May 2020 through an online survey platform (Qualtrics). On 23 Apr. 2020, we distributed both instructor and student surveys simultaneously to instructors. Instructors were responsible for administering student surveys in their classes and offering a small incentive (0.5% extra credit) for completion. Instructors did not receive compensation for completing instructor surveys. After the end of the semester, we provided instructors deidentified reports summarizing student survey results in their course(s).

### 3.3 Instrumentation

#### 3.3.1 Instructor survey

Our instructor survey included the following components: (a) basic information including the course title and number of students enrolled; (b) inventories of updates and accommodations provided during adaptation and remote teaching developed by the research team; (c) questions about the extent of video‐conferencing use, typical activities in synchronous video‐conferenced courses, and technologies used, generated by the research team; (d) selected items from Wieman and Gilbert's (2014) *Teaching Practices Inventory* (TPI) representing the structure of supplementary materials, assignments, and feedback in the course; (e) items modified from the TPI inventorying collaborative teaching approaches utilized during remote teaching; and (f) a brief validity check. In addition, after each section, instructors were asked to rate whether they perceived “positive,” “negative,” or “neutral” effects on this category during remote learning compared with previous semesters. Items consisted of rating scales, “select all” questions, and open‐ended questions.

In the brief validity check, 6 out of 10 instructors reported that the instructor survey “very” or “extremely” accurately and completely captured their teaching method and response to the pandemic on a 5‐point scale. Four instructors selected that the survey instrument was “slightly” or “moderately” accurate and complete in describing their teaching. In open‐ended responses, instructors elaborated that additional inquiry should more specifically address the needs of laboratory‐based and large enrollment courses and the time requirements for preparing online instructional materials.

#### 3.3.2 Student survey

We based assessments of students’ remote learning experience on the Community of Inquiry (CoI) framework scale developed by Arbaugh et al. (2008). The three‐factor scale corresponds to three facets of educational experience: social presence, cognitive presence, and teaching presence. Each CoI factor separates qualitatively into subscales (Table 3). Students rated the 34 items in the CoI questionnaire on a 5‐point scale from “strongly disagree” to “strongly agree.”

**TABLE 3:** Summary of categories and sub‐categories of student educational experience in the community of inquiry (CoI) framework

Category	Sub‐category
Social presence	Affective expression Open communication Group cohesion
Cognitive presence	Triggering event Exploration Integration Resolution
Teaching presence	Design and organization Facilitation of discourse Direct instruction

In addition to assessing students’ perceived educational experience during emergency remote learning, we evaluated two variables we considered more distal, interpretive outcomes: (a) satisfaction with emergency remote learning and (b) perceived change in engagement during emergency remote learning compared with prior in the semester. We assumed that both demographic and CoI educational experience variables would contribute to students’ reported satisfaction and change in engagement outcomes. Students rated their satisfaction with remote learning on a 5‐point Likert scale from “extremely dissatisfied” (1) to “extremely satisfied” (5). Students rated perceived change in engagement by selecting “more engaged,” “less engaged,” or “neutral,” and optionally elaborating in an open‐ended response. One member of the research team triangulated student responses to rating scales and open‐ended responses, showing excellent agreement.

### 3.4 Statistical analysis

We conducted all statistical analyses in R and declared significance at *p* < .05 (R Core Team, 2020). We computed summary statistics using base R and dplyr functions (Wickham, François, Henry, & Müller, 2020). We fit a confirmatory factor analysis (CFA) in lavaan (Rosseel, 2012). We retained only the first response for students who provided responses in multiple courses, leaving *N* = 261. Due to moderate skewness and kurtosis in teaching presence and cognitive presence variables, we used maximum likelihood estimation and robust Huber‐Sandwich estimation of standard errors (Huber, 1967; White, 1980). Fit indices included the scaled comparative fit index (CFI), the scaled Tucker‐Lewis index (TLI), the scaled root mean squared error of approximation (RMSEA), and the standardized root mean square residual (SRMR). The CFA indicated adequate reliability and validity of the community of inquiry questionnaire in our sample after allowing correlations between two item residuals within each factor (CFI = .89; TLI = .89; RMSEA = .06; SRMR = .06).

For regression modeling, our unit of analysis was student within course. Before fitting regression models, we prepared data in several steps. To avoid imbalance in random effect group sizes, we deleted responses from two courses that had two or fewer student respondents (courses I and J) leaving 257 responses. For parsimony, we dichotomized predictor variables major (animal and dairy science majors vs. non‐majors), gender (male vs. female and non‐binary/not‐specified), racial and ethnic identification (white vs. underrepresented minority), community type (rural vs. urban), and first‐generation college student (yes vs. no) and recoded with dummy contrasts based on reference groups suggested by the literature. Classification (freshman, sophomore, junior, senior, graduate/non‐traditional) was treated as a factor.

To fit regression models, we used the lme4 package (Bates, Maechler, Bolker, & Walker, 2015). Following the recommendation of Barr, Levy, Scheepers, and Tily (2013), we fit random effects structures with the maximum complexity justified by the data and experimental design. In most cases, this amounted to a random intercept for “course” to account for non‐independence. Due to modest sample size, we did not consider interactions between predictors (Heo & Leon, 2009). We retained all demographic and educational experience predictors in models regardless of significance due to their theoretical importance. We checked for multicollinearity of predictors and homoscedasticity of residuals by computing variance inflation factors and inspecting residual plots, respectively.

### 3.5 Qualitative analysis

To recover additional explanatory data, one researcher analyzed instructor and student qualitative responses using a rapid coding approach (Taylor, Henshall, Kenyon, Litchfield, & Greenfield, 2018). To protect the confidentiality of instructor participants, course and instructor information were removed from qualitative data before analysis. All instructor respondents and a fraction of student respondents (M = 14.5%, SD = 0.07% within the *n* = 8 courses modeled) provided qualitative data. Following Fereday and Muir‐Cochran's hybrid approach (Fereday & Muir‐Cochrane, 2006), analysis consisted of two stages. In the deductive stage, we applied a codebook based on the dependent variables in our conceptual model and summarized data into relevant categories. In the inductive stage, we re‐analyzed responses within categories without an a priori framework, searching for explanatory factors with practical relevance to stakeholders. After defining a posteriori codes, we applied these codes to the data to identify inductive themes within deductive categories. We paraphrased themes and selected exemplary quotes to present in the results section.

## 4 RESULTS AND DISCUSSION

### 4.1 Perceived preparedness

Most instructors (*n* = 6) rated their course as “not at all” or only “slightly” online‐ready prior to the Spring 2020 semester, reporting that some course materials and assignment submissions had been online prior to the Spring 2020 semester. Most instructors (*n* = 7) had no or little experience with online teaching before adapting their courses. Likewise, 72.6% of students reported having little to no experience with taking courses taught predominantly online.

Pre‐pandemic studies documented resistance toward remote education among faculty in colleges of agriculture and natural sciences (Boland, 2017; Roberts, Moore, & Dyer, 2005). Although educational technologies are increasingly central to higher education's value proposition, most instructors have limited experience teaching and learning in online courses (Horvitz, Beach, Anderson, & Xia, 2015; Marek, 2009). Research suggests that under typical conditions, online courses require more time to develop and implement compared with in‐person instruction, and this is especially true for instructors with little experience teaching remotely (Freeman, 2015). Still, even before the pandemic, distance education was growing rapidly as a mode of instruction in higher education. The National Center for Educational Statistics (NCES, 2019) reported that in 2018, roughly one‐third of students were enrolled in at least one remote learning course at U.S. post‐secondary institutions. Still, only 16.6% of students were enrolled in exclusively remote learning courses (NCES, 2019). This research is consistent with our finding that most students and instructors were relatively inexperienced with remote education and coped with an unprecedented challenge to adapt to the Spring 2020 emergency remote learning circumstances.

### 4.2 Remote teaching practices

#### 4.2.1 The adaptation process

Many instructors provided students with accommodations and support while adapting to online learning (Table 4). In an open‐ended response, one instructor expressed a preference for open, informal communication with students during the transition to remote learning, remarking that students were “already inundated with information” from other courses. Instructors used a variety of university‐licensed (Canvas, Blackboard Collaborate, Webex) and independent (Zoom, personal website, other) technologies to teach remotely. Instructors reported moderate satisfaction (dissatisfied, *n* = 1; neutral, *n* = 4; satisfied, *n* = 5) with the performance of these technologies during remote teaching.

**TABLE 4:** Instructors’ (*N* = 10) reported accommodations and technologies during emergency remote teaching in the Spring 2020 semester

Item	Freq.
Training and supports for students during adaptation to remote learning	
Grading policies were altered to account for the adjustment to online learning	6
Updated syllabus was posted	6
Students were provided online‐learning help resources (e.g., help navigating online learning environment)	4
Students were surveyed about their connectivity needs, access to internet	3
Students were provided well‐being resources (e.g., mental health support)	2
Offered that students should contact instructors with questions/feedback	2
None of the above/not applicable	2
Switched from discussion mode to lecture mode	1
Accommodation procedures during remote learning	
Accommodations made for students with unanticipated technical difficulties (e.g., internet or computer crashing)	8
Accommodations made for students with no ability to video‐conference (e.g., low internet speed)	7
Accommodations made on an as‐needed basis for individual students (not announced to students or added to syllabus)	6
All new accommodations announced VERBALLY	5
All new accommodations added IN WRITING to syllabus or in course materials	4
Accommodations made for students with additional child care/family care responsibilities	3
None of the above/not applicable	1
Technologies used during remote learning	
Canvas	9
BBCollaborate Ultra	8
Other/none of the options/not applicable	2
Zoom	2
Instructor's own course website	2
Webex	1
Google Hangouts	1
Adobe Connect	0
Piazza	0

Due to the unprecedented impacts of the COVID‐19 pandemic on students’ day‐to‐day lives, the literature provides relatively few descriptions of appropriate accommodations. Our results indicated that most instructors were flexible and accommodating to student needs during emergency remote learning, which Petillion and McNeill (2020) suggested aligns with student preferences. The majority of instructors used the dominant instructional technologies supported by the university, suggesting that institutional support plays a critical role in faculty technology adoption (Marek, 2009).

#### 4.2.2 In‐class engagement

Most instructors expected students to prepare for in‐class engagement during emergency remote learning (Table 5). Nine of 10 instructors reported that instructor‐created content (e.g., slides, worksheets, self‐authored papers) played the most central role in their courses, above instructor independent content (materials from external sources, not the instructor or students) and student‐created content (e.g., student projects, presentations, summaries). In an open‐ended response, one instructor remarked that the abrupt change to remote teaching necessitated a more instructor‐dominant approach but that they saw potential for more student‐centered remote teaching in future iterations.

**TABLE 5:** Count of animal sciences instructors (*N* = 10) employing selected remote teaching methods during the Spring 2020 semester

Item	Count
Students asked to read/view material for upcoming class session	8
Students read/view material on upcoming class session and complete assignments or quizzes on it shortly before class or at beginning of class	6
Reflective activity at end of class, e.g., “one‐minute paper” or similar (students briefly answering questions, reflecting on lecture and/or their learning, etc.)	4
Student presentations (verbal or poster)	3
None of the above/not applicable	1

The range of practices used by instructors in our sample suggests a continuum from traditional lecture‐based, instructor‐dominant instruction to flipped‐classroom, student‐centric approaches (Mok, 2014). In our sample, most instructors appeared nearer to the traditional lecture‐based approach, although instructor use of pre‐class and reflective assignments suggested some use of student‐centered learning strategies. Research has related assignments prompting metacognition, self‐guided inquiry, reflexivity, and interaction with students and instructors with student engagement and performance in online coursework (Garcia‐Vedrenne, Orland, Ballare, Shapiro, & Wayne, 2020; Gray & Diloreto, 2016; Kahn, Everington, Kelm, Reid, & Watkins, 2017; Vincent, Pilotti, & Hardy, 2016). However, building such assignments into coursework requires time, advance planning, and input from students—all of which instructors lacked during the Spring 2020 semester (Ramachandran & Rodriguez, 2020; Wurdinger, Wurdinger, & Allison, 2017). In non‐emergency situations, instructors can likely leverage student‐centric assignments to greater engagement and learning gains.

#### 4.2.3 Asynchronous and synchronous approaches

**FIGURE 2:**
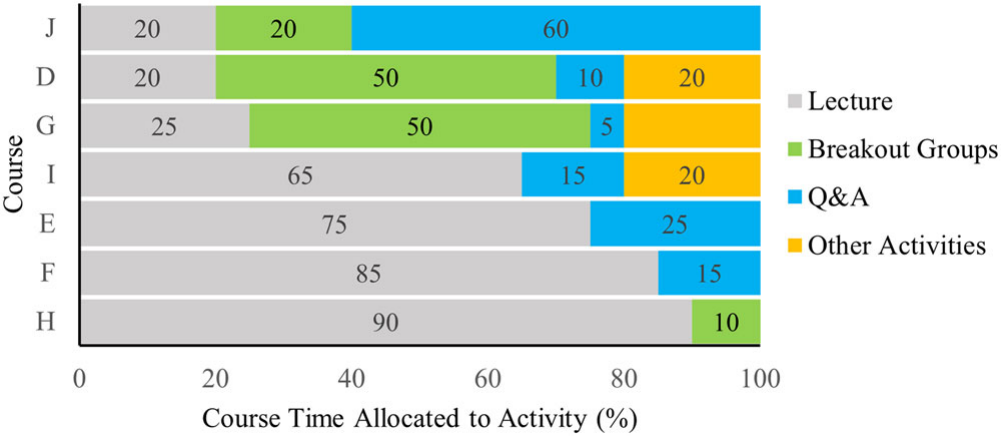
Allocation of video‐conferenced session time to selected activities in courses with synchronous components (*n* = 6 instructors, 7 courses)

Of the 10 instructors surveyed, 6 used synchronous video‐conferences to replace a significant portion of course activities, with the remainder posting narrated slide decks for students to view asynchronously. Several instructors using synchronous video‐conferences noted that the allocation of time for various synchronous activities in their course was moderately different during remote teaching than in previous semesters (*n* = 4). Approaches to video‐conferenced classes varied among instructors (Figure 2).

Results showed a dominance of instructor‐centric, lecture‐based teaching strategies during synchronous session in emergency remote learning. This is consistent with research describing typical university science teaching before the pandemic (Stains et al., 2018). Aside from two instructors who used a variety of student‐centered strategies in courses J, D, and G (Table 1), our results showed underutilization of strategies that engage students in synchronous sessions (McBrien, Cheng, & Jones, 2009). Additionally, results showed that few instructors chose to teach asynchronously, which may have stemmed from intentions to ease students’ transition to emergency remote instruction during the Spring 2020 semester. Although each strategy requires a drastically different approach, research has shown that asynchronous and synchronous instruction can achieve similar engagement, satisfaction, and learning outcomes for students compared with in‐person instruction (Neuhauser, 2002; Somenarain, Akkaraju, & Gharbaran, 2010). However, regardless of teaching approach, lecture‐based instruction typically produces lower engagement and learning compared with student‐centered techniques (Erickson, Marks, & Karcher, 2020; Freeman et al., 2014). As Jeffery and Bauer (2020) suggest, building capacity for remote learning in the long‐term will require more substantial departmental and institutional support for implementing student‐centered instruction.

Table 6 summarizes the use intensity of various video‐conferencing features by instructors teaching synchronous video‐conferenced classes. All instructors reported using the chat box in nearly every class. Instructors reported using Google Docs or other synchronous workspaces, polls, breakout groups, and virtual whiteboards less frequently or not at all. In open‐ended responses, instructors shared limitations associated with the chat box (too distracting), the breakout rooms (inability to pre‐assign groups), and the virtual whiteboard (difficult to draw smoothly).

**TABLE 6:** Frequency of instructors’ (*n* = 6) use of selected technologies during video‐conferenced synchronous classes

Item	Never	A few classes	Nearly every class
Chat box	0	0	6
Google docs or another synchronous workspace	3	1	2
Polls	2	3	1
Breakout groups	3	2	1
Virtual whiteboard	2	3	1

#### 4.2.4 Supporting materials

Table 7 describes the supplemental materials available to students during remote teaching. Nearly all instructors reported providing lecture notes or recorded slide decks. Half or fewer indicated providing additional resources such as articles from related academic literature, grading rubrics, discussion boards, worked examples, and examples of exemplary projects. The majority of instructors (*n* = 7) stated that their use of supplemental materials was “about the same” during remote teaching compared with prior in‐person semesters. The remaining instructors reported perceiving either positive (*n* = 1) or negative (*n* = 2) effects of remote instruction on their use of supplemental materials.

**TABLE 7:** Count of instructors (*N* = 10) providing selected types of supporting materials during remote learning in the Spring 2020 semester

Item	Count
Lecture notes or course PowerPoint presentations (partial/skeletal or complete)	9
Animations, video clips, or simulations related to course material	5
Other instructor‐selected notes or supporting materials, pencasts, etc.	5
Articles from related academic literature	5
Grading rubrics for papers or large projects	5
Solutions to homework assignments	4
Student wikis or discussion boards with little or no contribution from you	3
Student wikis or discussion boards with significant contribution from you or TA	3
Worked examples (text, pencast, or another format)	3
Practice or previous year's exams	3
Examples of exemplary papers or projects	1
None of the above/not applicable	0

In recent studies, many instructors adopted multipronged, multimodal teaching strategies to avoid inequities due to student circumstances (e.g., connectivity) during the Spring 2020 semester (Czerniewicz et al., 2020). Our results showed that most instructors provided a range of supplementary materials to accompany synchronous or asynchronous course sessions, although instructors did not report substantial changes in supplementary materials following the pandemic. In the TPI, Wieman and Gilbert (2014) suggest that in general, providing more and higher quality supporting materials is associated with greater student success. In non‐emergency situations with more time for advance preparation, natural sciences educators may have adequate time and resources to enhance the supporting materials provided to students for remote instruction.

#### 4.2.5 Assignments and feedback

Table 8 describes the structure of assignments and feedback in remote courses. The majority of instructors assigned regular graded homework or problem sets at intervals of 2 weeks or less. No instructors reported using homework or problem sets that did not contribute to course grades. Fewer than half of the courses involved student‐driven papers or projects or group assignments. Most instructors (*n* = 8) indicated that the quality of students’ assignments was about the same during remote teaching as during previous semesters. However, one instructor who reported a decline in the quality of assignment submissions associated with remote learning opined that a lack of structure with remote teaching caused students to approach coursework less systematically.

**TABLE 8:** Structure of assignments and feedback to students provided by *N* = 10 animal sciences instructors during remote teaching in the Spring 2020 semester

Item	Count
Assignments during remote teaching	
Homework/problem sets assigned and contributed to course grade at intervals of 2 weeks or less	7
Paper or project (an assignment taking longer than 2 weeks and involving some degree of student control in choice of topic or design)	4
Encouragement and facilitation for students to work collaboratively on their assignments	4
Group projects or assignments	3
None of the options/not applicable	2
Homework/problem sets assigned or suggested but did not contribute to course grade	0
Feedback during remote teaching	
Students see graded midterms	8
Students see graded assignments	6
Students see assignment answer key	6
Assignments with feedback	5
Students see midterm exam quiz answer key	4
Students explicitly encouraged to meet individually with instructor	4
Online office hours offered	4
None of the above/not applicable	0

Regarding feedback to students, most instructors indicated providing graded midterms and graded assignments for students to review (Table 8). A minority of instructors offered online office hours or explicitly encouraged students to meet with them. Although six instructors indicated that feedback in the course was “about the same” as prior semesters, another three mentioned that remote teaching hindered their ability to provide students with feedback. In open‐ended responses, one instructor mentioned that students “respond better in an in‐person meeting for dialogue about study/learning challenges.” Another suggested that they “couldn't engage with students before or after class…and mentor students.”

The nature and frequency of interactions with faculty and other students shape undergraduates’ personal, social, and academic outcomes (Cotten & Wilson, 2006). At present, it is unclear how emergency remote teaching affected instructors’ interactions with students in and out of class, although pre‐pandemic research found that students’ use of virtual and in‐person office hours was similar (Li & Pitts, 2009). Indeed, virtual feedback systems may be preferable to in‐person systems for certain students (Kelly, Keaten, Hazel, & Williams, 2010). Wieman and Gilbert (2014) recommend that more frequent, more collaborative assignments and more feedback from instructors are associated with improved student outcomes.

#### 4.2.6 Learning how to teach remotely

Table 9 shows instructors’ self‐reported strategies used to transition to remote teaching. Most instructors discussed the process of adapting courses to remote and remote teaching practices with colleagues. Many described attending university‐ and/or corporate‐sponsored training for remote teaching, and half reported consulting the literature. A minority of instructors indicated that they had turned to blogs, websites, or other informal resources. No instructors reported sitting in on colleagues’ classes to learn ideas. Importantly, several courses were co‐taught with other faculty (*n* = 3), undergraduate student teaching assistants (TAs; *n* = 3), and graduate student TAs (*n* = 4) such that the instructor felt a shared responsibility to adapt to remote instruction (*n* = 3). Nearly all instructors (*n* = 9) reported that their collaborative teaching efforts were “about the same” during remote teaching as with earlier semesters. The remaining instructor felt remote teaching positively affected their collaborative teaching.

**TABLE 9:** Count of animal sciences instructors (*N* = 10) employing select strategies to learn how to teach their course remotely in the Spring 2020 semester

Item	Count
Discussed how to adapt elements of the course to online format with colleague(s)	7
Discussed online teaching practice with colleague(s)	7
Participated in additional training offered for instructors (e.g., training to use Canvas, BBCollaborate, other continuity of instruction resources).	6
Read literature about teaching and learning relevant to moving the course online	5
Used or adapted materials provided by colleague(s)	4
Read blogs, websites, or other informal resources relevant to moving the course online	4
None of the options/not applicable	1
Sat in on colleague's class (any class) to get/share ideas for teaching	0

Our results indicate that instructors drew from a diversity of sources to adapt to emergency remote teaching but did not seek support from colleagues or the broader teaching community to a great extent. This is consistent with reported pre‐pandemic behavior of instructors in our sample and with research showing that faculty typically collaborate to a lesser extent on teaching than on research or service activities (Joseph, Oh, & Ackerman, 2018; Ramsden, 1998). In non‐emergency settings, faculty collaboration in scholarship of teaching and learning or peer support networks has been shown to develop pedagogical knowledge, improve technical competencies, and facilitate sharing of resources (Roxå, Olsson, & Martensson, 2008; Erickson et al., 2020; Kyei‐Blankson, Keengwe, & Blankson, 2009). In the long term, natural science educators can accelerate the development of remote and blended instruction by expanding and strengthening teaching collaborations. Critically, institutions and departments must create teaching culture and support systems to unlock the capacity‐building benefits of collaborative teaching (Wingo, Ivankova, & Moss, 2017).

### 4.3 Student educational experience

Table 10 shows student self‐rated educational experience based on the Community of Inquiry framework questionnaire (Arbaugh et al., 2008). Responses centered at the upper range of the scale for all subscales. The high mean observed across CoI subscales in our sample during emergency remote learning is comparable to values achieved in typical online courses (Díaz, Swan, Ice, & Kupczynski, 2010; Kozan & Richardson, 2014). This result was unexpected considering the inexperience of instructors in our sample with remote teaching, and previous research that showed the degree of instructor experience with online teaching has a significant positive correlation with teaching presence and cognitive presence (Arbaugh, 2008).

**TABLE 10:** Student perceptions of educational experience based on community of inquiry framework subscales ranked according to means (*N* = 261 responses)

Subscale	Items	Category	α^a^	M	SD
Design and organization	4	Teaching presence	.85	4.4	0.8
Resolution	3	Cognitive presence	.85	4.2	1.0
Direct instruction	3	Teaching presence	.76	4.1	1.0
Facilitation	6	Teaching presence	.89	4.1	1.0
Integration	3	Cognitive presence	.82	4.0	1.0
Exploration	3	Cognitive presence	.78	3.8	1.1
Open communication	3	Social presence	.83	3.8	1.1
Triggering event	3	Cognitive presence	.89	3.7	1.2
Group cohesion	3	Social presence	.82	3.6	1.1
Affective expression	3	Social presence	.84	3.3	1.2

^a^ Cronbach's alpha coefficient for the subscale.

Cronbach's alpha coefficients corroborated the reliability of subscales (Arbaugh, Bangert, & Cleveland‐Innes, 2010). Notably, greater means were associated with teaching presence and cognitive presence subscales compared with the social presence subscales. This is consistent with instructors’ reported instructor‐dominant teaching practices. The highest‐rated subscale, design and organization, indicated that students in our sample perceived that instructors very clearly communicated course topics, course goals, due dates, and instructions. High ratings on resolution suggested that students perceived that the course developed their abilities to apply knowledge learned. Conversely, students rated social presence subscales group cohesion and affective expression nearer to neutral. Neutral affective expression indicated that participants may not have felt a sense of belonging in the course or experienced positive interactions with other course participants (Garrison & Aykol, 2013). Neutral group cohesion indicates that participants may not have developed a sense of collaboration, trust, or respect through participation in the course. In our sample, subscales with lesser means generally showed greater standard deviations, which may represent actual differences or the artifactual ceiling imposed by Likert scale response options.

Table 11 presents linear mixed‐effects regressions with CoI scales social presence, cognitive presence, and teaching presence as dependent variables, demographic predictors as fixed effects, and course as a random intercept. We found no demographic predictors significantly predicted CoI scale variables during emergency remote learning. This is in contrast with past research in which the demographics such as ethnicity, gender, and discipline significantly impacted CoI variables in online courses (Dempsey & Zhang, 2019; Wicks, Craft, Mason, Gritter, & Bolding, 2015) and past research showing demographic differences in students’ adaptability to online learning (Xu & Jaggars, 2013). It is plausible that our sample lacked the diversity and sample size to make differences apparent, or that the accommodations and multimodal support provided by instructors created equitable learning environments.

**TABLE 11:** Linear mixed‐effects models describing variance in Community of Inquiry scales attributable to student demographics and course

	Social presence		Cognitive presence		Teaching presence	
Predictors	Est.	*p*	Est.	*p*	Est.	*p*
(Intercept)	2.72	<.001	2.73	<.001	3.06	<.001
Non‐major	0.10	.55	0.17	.25	0.08	.57
Non‐male	–0.17	.15	0.07	.53	0.04	.69
First‐generation	0.18	.16	0.03	.80	0.05	.61
Urban	0.09	.44	0.11	.33	0.05	.60
Minority	0.12	.35	0.01	.94	0.05	.65
Sophomore	–0.07	.66	0.08	.62	0.10	.46
Junior	–0.17	.34	0.01	.97	0.04	.79
Senior	–0.18	.30	0.15	.34	0.14	.35
Graduate/non‐traditional	0.65	.20	0.55	.25	0.24	.58
Random effects^a^						
σ^2^	0.67		0.58		0.49	
τ_00 course_	0.19		0.12		0.10	
ICC _course_	.22		.17		.17	
*N*_course_	8		8		8	
Observations	257		257		257	
Marginal *R* ^2^	.05		.03		.01	
Conditional *R* ^2^	.26		.20		.18	

^a^ The σ^2^ and τ_00_ represent the within‐group and between‐group variance, respectively. ICC _course_ shows the intraclass‐correlation coefficient for the random effect of course.

In our study, restricted maximum likelihood (REML)‐based likelihood ratio model comparisons indicated that the random intercepts for course explained a significant proportion of the variance in CoI scales (*p* < .001). The marginal coefficient of determination (*R* ^2^) shows the variance attributable to only the fixed effects, whereas the conditional *R* ^2^ shows the variance attributable to both fixed and random effects (West, Welch, & Galecki, 2014). Intra‐class correlation coefficients (ICC) show the within‐course similarity of CoI variables. Results presented in Table 11 suggest that instructor and student factors specific to the course influenced social presence, cognitive presence, and teaching presence in our sample. Our study is the first to our knowledge to report variance component estimates describing students’ educational experience in remote courses within a single university department. However, our results were consistent with Wilson, Summers, and Wright (2020) in which multi‐level modeling showed significant faculty impacts on student educational experience in seven in‐person courses within a single university department. In line with past research on in‐person instruction, our findings showed that instructors significantly impact student perceptions of the classroom cognitive presence, social presence, and teaching presence, which has implications for student educational experience and satisfaction outcomes (Burgess, 2018; Umbach & Wawrzynski, 2005).

In open‐ended responses, students discussed ways in which remote learning affected their educational experience. For social presence, several submitted that their emergency remote learning courses lacked opportunities for interaction, for example, “the lectures were recorded which made it difficult to ask questions…I expect to have an opportunity to converse with my professor during class time,” and “It was more difficult to understand what was being emphasized within presentations without being able to see the professors’ faces.” Other students discussed the difficulty of engaging in discussions with peers online (particularly when all videos are turned off) and holding peers accountable virtually. For cognitive presence, students mentioned difficulties self‐motivating, focusing, and keeping up with scheduled assignments. For teaching presence, students shared appreciation for instructors who communicated regularly, used technology seamlessly, established accommodation policies, created accountability structures, organized course materials, and provided multimodal learning resources. Several students described competing interests (employment and family), internet connectivity issues, and mental health concerns further affecting their educational experience. Conversely, one student who described commuting to class prior to the pandemic noted that remote learning “saved me time and stress.” Although no demographic variables significantly predicted quantitative engagement metrics across our student sample, qualitative results reinforce that certain students felt their educational experiences were shaped by non‐academic factors during emergency remote learning (Petillion & McNeill, 2020; Ramachandran & Rodriguez, 2020).

### 4.4 Student satisfaction and engagement outcomes

#### 4.4.1 Student satisfaction with emergency remote learning

Table 12 presents a linear mixed‐effects regression relating students’ satisfaction with emergency remote learning to predictors representing student personal information and course perceptions. On average, students reported being “neither satisfied nor dissatisfied” with emergency remote learning (M = 3.4, SD = 1.1). Meta‐analytical research has shown that student satisfaction may be reduced for synchronous and asynchronous remote courses compared with in‐person courses (Ebner & Gegenfurtner, 2019; Lowenthal, Bauer, & Chen, 2015). However, the particular factors causing differences in satisfaction are unclear. At this stage, we included demographic variables to control for direct effects outside of CoI variables and found no significant associations. However, perceived social presence had a strong positive influence on student satisfaction. These results are consistent with past research showing that social presence strongly predicts learners’ satisfaction with remote learning as a learning modality (Arbaugh & Benbunan‐Fich, 2007) and satisfaction with remote courses (Choy & Quek, 2016; Lee, Hoe Looi, Faulkner, & Neale, 2020). This may reflect the larger variance in social presence relative to cognitive presence and teaching presence in our sample. Our study assumed that students’ perceived CoI experience in a course, unmeasured variables, and their interpretations would contribute to their satisfaction with emergency remote learning. However, the large amount of variance explained by the CoI variables relative to the variance attributed to the random effect of course indicates that the CoI variables explained most course‐related variance in satisfaction in our sample. This result suggested that the CoI is a promising framework to capture differences in instructor‐ and course‐level variation relevant to student‐reported satisfaction at the end of the semester.

**TABLE 12:** Linear mixed‐effects regression describing student satisfaction with emergency remote learning from selected educational experience and demographic variables

	Satisfaction with emergency remote learning		
*Predictors*	Estimates	95% CI^a^	*p*
(Intercept)	0.66	0.00–1.31	.05
Teaching presence	0.23	–0.05–0.51	.10
Social presence	0.37	0.18–0.55	<.001
Cognitive presence	0.22	–0.06–0.50	.13
Non‐major	0.13	–0.17– 0.44	.39
Non‐male	0.25	–0.01–0.52	.06
First‐generation	–0.15	–0.42–0.13	.29
Urban	0.20	–0.07–0.46	.15
Minority	–0.18	–0.47–0.11	.22
Sophomore	–0.23	–0.59–0.13	.20
Junior	0.27	–0.09–0.64	.14
Senior	0.15	–0.21–0.50	.42
Graduate/non‐traditional	0.28	–0.83–1.40	.62
Random effects^b^			
σ^2^	0.85		
τ_00_ _course_	0.02		
ICC _course_	.03		
*N*_course_	8		
Observations	257		
Marginal *R* ^2^	.32		
Conditional *R* ^2^	.34		

^a^95% Confidence interval for the estimate; ^b^The σ^2^ and τ_00_ represent the within‐group and between‐group variance, respectively. ICC _course_ shows the intraclass‐correlation coefficient for the random effect of course.

A dominant theme related to satisfaction in student open‐ended responses described students adjusting expectations. For example, students adjusted expectations to match the course's pre‐pandemic format, for example, “[this] wasn't a course that had an abundance of student–professor or student–student interaction in the first place”; to match perceived limits of remote learning, for example, “my dissatisfaction with remote learning is not due to my professors in any way and I do not know what they could have done to make it better”; and to match experiences in other courses in which they were currently enrolled, for example, “I felt that this class had the smoothest transition to online learning out of all the classes I am in this semester.” Conversely, other students expressed dissatisfaction in relation to unadjusted expectations, for example, “I was really looking forward to the labs associated with this course…I feel a bit robbed of the experience,” “I feel conned out of thousands of dollars, and cheated out of what could have been a fantastic class,” and “If I found it more enjoyable and engaging I would have paid to go to an online school.” Several students showed empathy toward instructors, recognizing their substantial efforts to adapt instruction in adverse circumstances. Taken together, quantitative and qualitative data related to satisfaction suggest that both educational experiences and students’ interpretations shape satisfaction with emergency remote learning. Perceived social presence exerted a strong positive influence on student satisfaction; however, students adjusted expectations using various reference points. Given the uncertainty at many institutions surrounding “the new normal,” varying expectations may continue to convolute student satisfaction with remote learning in future semesters. As Hodges et al. (2020) suggest, early experiences and interpretations with remote learning have implications across remote, blended, and online learning.

#### 4.4.2 Student‐perceived change in engagement during emergency remote learning relative to prior

Table 13 shows a generalized linear regression with a logit link describing the relative risk of students reporting losing engagement during emergency remote learning. Of 257 student respondents, 134 (52.1%) reported a negative effect of emergency remote learning on their engagement compared with earlier in the semester, whereas 113 (44.0%) and 10 (3.9%) reported neutral and positive effects, respectively. This is consistent with the expectation that traumatic, unexpected changes have negative impacts on student engagement (Wang et al., 2020). Although we first fit a mixed model to describe change in engagement, this produced a singular fit. Per Barr et al. (2013), we removed the random term to allow a non‐singular fit and estimated a logit‐link generalized linear model. At this stage, we included demographic variables to control for direct effects outside of CoI educational experience variables and none significantly predicted the odds of losing engagement. However, each point increase in perceived social presence was associated with significantly reduced odds of reporting losing engagement during emergency remote learning. Teaching presence was a marginally significant predictor of reduced odds for losing engagement. These results again reinforced the explanatory power of the CoI framework for distal outcomes such as engagement and satisfaction, and the importance of social presence in remote learning educational experience (Khalid & Quick, 2016; Lee et al., 2020).

**TABLE 13:** Generalized linear regression with logit link illustrating the relative risk of students reporting losing engagement during emergency remote learning for selected educational experience and demographic variables

Losing engagement during emergency remote learning^a^			
Predictors	Risk ratio	CI	*p*
(Intercept)	1.7	1.4–1.9	<.001
Teaching presence	0.7	0.4–1.0	.06
Social presence	0.8	0.6–1.0	<.05
Cognitive presence	1.1	0.8–1.4	.44
Non‐major	1.1	0.8–1.4	.35
Non‐male	0.9	0.7–1.2	.73
First‐generation	1.2	0.9–1.5	.12
Urban	0.9	0.6–1.2	.50
Minority	0.8	0.5–1.1	.18
Sophomore	1.2	0.8–1.6	.31
Junior	1.1	0.7–1.5	.57
Senior	1.3	0.9–1.6	.19
Graduate/non‐traditional	1.7	0.5–2.1	.23
Observations	257		
Tjur's *R* ^2^	.10		

^a^ Risk ratios, 95% confidence intervals (CI), and *p* values were calculated from the log‐odds coefficients.

Nearly one‐third of open‐ended responses represented a theme we termed “student beliefs about engagement in remote learning.” Overwhelmingly, students shared that they believed remote learning to be a less‐engaging modality compared with in‐person. One student suggested that they required physical classroom attendance to feel engaged. Several issued judgments on the unsuitability of particular courses to remote learning. In open‐ended responses, student beliefs about remote learning appeared independent of educational experiences, for example, “[My professor] did a great job of adjusting. Online learning simply doesn't work well for me.” Only one respondent demonstrated reflexive awareness that “It is not ideal to be forced to take online classes when you are used to in‐person instruction.” Based on open‐ended data, negative beliefs about engagement in remote learning may represent a substantial hurdle in creating engaging remote educational experiences (Xie & Huang, 2014). Although we did not investigate instructor beliefs and attributions, research suggests that instructors’ mindsets influence teaching practices and may also be an important topic for research (Aragón, Eddy, & Graham, 2018).

## 5 LIMITATIONS

Our study has at least four important limitations. First, we used a convenience sample representing a limited group of instructors and students within a single department at a single university. A fraction of our target sample did not provide responses to surveys. Inference outside our population will require future meta‐analytic work or cross‐sectional research with more advanced sampling designs. Second, we relied on self‐report measures of teaching practices and student educational experience (Douglass, Thomson, & Zhao, 2012). To avoid potential biases of self‐report data, future researchers might capitalize on the richness of behavioral data captured through learning management systems and in course recordings (Wichadee, 2014). Third, our research is observational and does not prove causal links among variables studied. We recommend future experimental work manipulating teaching practices or CoI variables in varying contexts (Oncu & Cakir, 2011). Fourth, we surveyed students and instructors during a disruptive semester and used theoretical frameworks and instrumentation developed prior to the pandemic (Wang et al., 2020). At present, it is unclear to what extent Spring 2020 semester patterns are comparable to pre‐pandemic studies or research developed in the later stages of adaptation.

## 6 CONCLUSIONS

As natural sciences instructors adapt to mid‐pandemic and post‐pandemic teaching, our results provide evidence that instructors with limited remote teaching experience can create equitable remote learning environments fostering social presence, cognitive presence, and teaching presence—even amid challenging global and institutional circumstances. In our study of emergency remote learning in Spring 2020, social presence varied the most between courses and predicted student outcomes of satisfaction and perceived change in engagement relative to in‐person instruction. Open‐ended responses revealed how students’ individual experiences were affected by expectations and beliefs about remote learning. Our cross‐sectional, self‐report study assessed a limited population of instructors and students in an animal and dairy science department during a disruptive semester. In the long term, more research is needed to develop mid‐pandemic and post‐pandemic natural science pedagogies that satisfy student needs in varying institutional and departmental contexts.

## RECOMMENDATIONS

Our results suggest the following actions for natural science educators teaching remote or hybrid courses:

- Surmount new challenges by relying on a community of colleagues with experience, if not expertise, in remote teaching and learning.
- Build social presence by crafting spaces for participatory learning, authentic self‐expression, and interpersonal interactions.
- Engage students cognitively by offering them multiple ways to learn (multi‐modal teaching) and plentiful supporting resources.
- Maintain strong teaching presence by establishing clear goals, policies, and accommodations for the course.
- Be conscious of instructor and student beliefs and expectations surrounding remote learning and confront any that detract from learning and satisfaction.

## AUTHOR CONTRIBUTIONS

M.G. Erickson: Conceptualization; Data curation; Formal analysis; Investigation; Methodology; Project administration; Validation; Visualization; Writing‐original draft; Writing‐review & editing. M.A. Wattiaux: Conceptualization; Formal analysis; Funding acquisition; Investigation; Methodology; Project administration; Resources; Supervision; Writing‐review & editing.

## CONFLICT OF INTEREST

The authors declare no conflict of interest.
